# Activation of CD4+ and CD8+ T-lymphocytes by insulin and GAD in patients with type 1 or 2 diabetes mellitus

**DOI:** 10.1530/EC-17-0230

**Published:** 2017-10-06

**Authors:** Borros Arneth

**Affiliations:** Institute of Laboratory Medicine and PathobiochemistryMolecular Diagnostics, University Hospital of the Universities of Giessen and Marburg, UKGM, Justus Liebig University, Giessen, Giessen, Germany

**Keywords:** type 1 diabetes mellitus, type 2 diabetes mellitus, CD4+ T-lymphocytes, CD8+ T-lymphocytes, autoimmunity, autoantibodies, insulin, GAD

## Abstract

**Background:**

The origin of autoimmune disease type 1 diabetes is still unknown.

**Aim:**

This study assessed the activation of CD4+ and CD8+ T-lymphocytes by human insulin and human glutamate decarboxylase (GAD) in patients with type 1 or type 2 diabetes mellitus (DM) and healthy volunteers.

**Materials and methods:**

The expression of CD69, a marker of T-lymphocyte activity, was determined in whole blood samples by flow cytometry after 12 h of incubation with or without insulin or GAD. The analysis included samples from 12 type 1 DM patients, 14 type 2 DM patients and 12 healthy volunteers.

**Results:**

Significant increases in the number of activated CD4+ and CD8+ T-lymphocytes following pre-incubation of whole blood samples with human insulin or GAD were observed in samples from patients with type 1 DM, whereas no activation of these cells was detected in samples from either type 2 DM patients or healthy subjects.

**Discussion:**

These results indicated that latent pre-activation of CD4+ and CD8+ T-lymphocytes in response to insulin or GAD epitopes occurred in type 1 DM patients.

**Conclusion:**

These findings suggest that pre-immunization against insulin and/or GAD might be associated with the development of type 1 DM. Alternatively, these results might reflect a non-specific, bystander autoimmune response.

## Introduction

Type 1 diabetes mellitus (DM) is an autoimmune disease in which T-lymphocytes attack insulin-producing beta cells in the pancreas (1). During the later stages of this progressive disease, pancreatic beta cells are massively reduced and sometimes nearly absent, leading to the severe-to-complete insulin deficiency characteristic of type 1 DM.

It remains unknown whether immunization against insulin occurs in patients with type 1 DM and whether this phenomenon can be detected in whole blood samples from these patients (2, 3). Notably, several reports have described immunization against glutamate decarboxylase (GAD), and anti-GAD antibodies are used to diagnose early type 1 DM.

Immunization against insulin and/or GAD might be associated with early pre-activation of T-lymphocytes. Indeed, autoantibodies specific to both insulin and GAD have been detected in patients at or prior to the onset of type 1 DM (2, 3, 4). However, it is unclear whether autoimmune activation continues during the later stages of this disease. In addition, it is not known whether this reactivity is limited to B-cells or whether it also occurs in T-cells.

To investigate this phenomenon in T-lymphocytes, whole blood samples from type 1 DM patients, type 2 DM patients and healthy subjects were incubated overnight with insulin or GAD. Then, aliquots of the whole blood samples were analyzed by flow cytometry, and the proportions of activated CD4+ and CD8+ T-lymphocytes were analyzed.

## Materials and methods

### Patients

During routine examinations, blood samples were obtained from 12 type 1 DM patients, 14 type 2 DM patients and 12 healthy volunteers. All blood samples were collected at 07:00 h, prior to administration of any medication (including insulin for the type 1 DM patients). The blood was collected in 8-mL ethylene diamine tetra-acetic acid collection tubes. The type 1 DM patients fasted for 9 h and were not administered any insulin for 12 h prior to blood collection. This study was approved by the ethics committee of University Mainz (Johannes Gutenberg University Mainz, Mainz, Germany). All methods were performed in accordance with the relevant guidelines and regulations. Diagnosis of type 1 or type 2 DM was performed according to the diagnostic criteria of the American Diabetes Association. For an overview of the patients’ and healthy subjects’ characteristics, please see Table 1 and Table 2. All subjects and patients provided informed consent to participate in this study.

**Table 1:** Characteristics of the patients and healthy subjects.

**Patients**	**Mean age** (years)	**Mean BMI**	**Mean disease duration** (years)	**Therapy**	**Autoantibodies at disease onset**	**Autoantibodies at time of study**
DM type 1 *N* = 12	34	23	3	Insulin	4 IAA/8 GAD	0 IAA/4 GAD
DM type 2 *N* = 14	65.9	30.4	8	Biguanide, sulfonyl-ureas	0 IAA/ 0 GAD	0 IAA/0 GAD
Healthy subjects *N* = 12	35	24	0	No therapy	0 IAA/0 GAD	0 IAA/0 GAD

**Table 2:** Effect of GAD on the CD64 activation status of CD4 and CD8 T-cells and comparison with the antibody status of the patients.

**GAD**	**CD69+**	**CD69+**		**Anti-GAD**	**Anti-GAD**
CD4	With	Without	Difference	At onset	At study
	19.5	6	13.5	Pos	Neg
	20.2	7.8	12.4	Pos	Neg
	15.84	4.6	11.24	Pos	Neg
	18.9	3.5	15.4	Pos	Neg
	22.5	8.2	14.3	Pos	Pos
	27.6	6.5	21.1	Pos	Pos
	25.3	5.8	19.5	Pos	Pos
	21	5.6	15.4	Pos	Pos
	6	6	0	Neg	Neg
	5	5	0	Neg	Neg
	6.2	6.3	−0.1	Neg	Neg
	5.4	5.5	−0.1	Neg	Neg

**GAD**	**CD69+**	**CD69+**		**Anti-GAD**	**Anti-GAD**
CD8	With	Without	Difference	At onset	At study
	15.6	6.1	9.5	Pos	Neg
	21.7	7.5	14.2	Pos	Neg
	19.1	5.7	13.4	Pos	Neg
	14.3	2.9	11.4	Pos	Neg
	17	7.2	9.8	Pos	Pos
	18.3	5.9	12.4	Pos	Pos
	22.9	5.4	17.5	Pos	Pos
	17.5	4.9	12.6	Pos	Pos
	6.4	6.4	0	Neg	Neg
	6	6	0	Neg	Neg
	6.3	6.4	−0.1	Neg	Neg
	5.6	5.7	−0.1	Neg	Neg

Anti-GAD at onset, GAD antibody status at the disease onset; anti-GAD at study, GAD antibody status at the time of the study; with, sample measured after pre-incubation with GAD; without, sample measured without pre-incubation with GAD.

### Lymphocyte activation

Lymphocytes were stimulated with whole blood by overnight incubation, and flow cytometry and quantification of CD69 on CD4+ and CD8+ T-lymphocytes were performed by FastImmune assay (BD Biosciences, Heidelberg, Germany), as previously described by Arneth (5, 6). Each blood sample was divided into 4 1-mL aliquots (Tubes 1, 2, 3 and 4). Tube 1 was treated with 50 µL of 0.2 g/mL concanavalin A (ConA; Sigma-Aldrich), Tube 2 was not treated, Tube 3 was treated with 50 IU/mL human insulin (Huminsulin; Eli Lilly) and Tube 4 was treated with human GAD (Sigma-Aldrich). In addition, the 12 samples from the healthy volunteers were treated using a BioTrek Transfection Kit (Stratagene) and subsequently incubated.

### Incubation period

Human whole blood samples (1000 µL) were incubated with 50 µL of 1000 IU/mL human insulin (50 IU Huminsulin; Eli Lilly) or 50 µL of 10 µg/mL GAD (Sigma-Aldrich). All tubes were gently mixed prior to incubation.

All blood samples were incubated for 14 h at 37°C (98.6°F). Once every two hours during the incubation period, the samples were carefully mixed to re-suspend the cells, thereby ensuring for adequate insulin exposure by redistribution of insulin throughout the samples. After overnight incubation, a 50-µL aliquot was obtained from each tube of whole blood and analyzed by flow cytometry.

### Cell staining

A commercially available assay (FastImmune Kit; BD Bioscience) was used for the flow cytometry analysis.

After overnight incubation, the cells were stained according to the manufacturer’s instructions (FastImmune Kit; Becton Dickinson; San Diego, CA, USA) with an antibody mixture containing fluorescently labeled antibodies against CD3, CD4, CD8 and CD69, which are extracellular epitopes that can be analyzed by flow cytometry without destroying cells.

Two microliters of the antibody mixture were added to a 50-µL aliquot of each human whole blood sample. As previously described by Arneth, CD3 is used to define the T-lymphocyte population, CD69 is an ‘early activation marker’ used to detect early activated T-lymphocytes, CD4 is the definitive T-helper marker (CD4+ T-lymphocytes) and CD8 is the definitive cytotoxic marker (CD8+ T-lymphocytes) (6). The anti-CD69 antibody used in the present study was labeled with R-phycoerythrin (PE), the most sensitive fluorochrome available for staining cells in this context. In addition, the antibodies specific to CD8, CD3 and CD4 were labeled with fluorescein isothiocyanate (FITC), peridinin chlorophyll protein (PerCP) and allophycocyanin (APC), respectively. The 50-µL whole blood samples were incubated with the antibody mixture for 20 min at room temperature, according to the manufacturer’s instructions.

### Erythrocyte lysis

As previously described by Arneth, before incubation of the erythrocytes with the antibody mixture for 20 min, the cells (in a total volume of 52 µL) were lysed with 450 µL of 1× lysis buffer, which was prepared according to the manufacturer’s instructions (Becton Dickinson) (6). After a second 20-min incubation with lysis buffer, the samples were analyzed by flow cytometry.

### Flow cytometry

The flow cytometry procedure has been previously described by Arneth (6). All samples were analyzed using a Becton Dickinson flow cytometer (BD FACSCalibur; Becton Dickinson). The FITC, PerCP, APC and PE fluorescence intensities were simultaneously measured and recorded, and the results were graphically evaluated. The cells of interest, T-lymphocytes, were gated based on the CD3 PerCP signal and side scatter, and the CD3+ T-lymphocytes were identified as the CD3+ population. For each sample, 30,000 events in the T-lymphocyte gate were recorded, and the T-lymphocyte gate was defined by plotting side scatter vs CD3 fluorescence on the *x*-axis.

CD4+ and CD8+ T-lymphocytes were distinguished based on the *x*-axis values in 2 separate plots. Expression of the early activation marker CD69 was plotted on the *y*-axis vs the number of either CD4+ or CD8+ T-cells on the *x*-axis in the plots (CD69 vs CD4 and CD69 vs CD8).

From these 2 plots, the proportions of activated CD4+ and activated CD8+ T-lymphocytes were determined as the percentages of double-positive CD4+/CD69+ T-lymphocytes and double-positive CD8+/CD69+ T-lymphocytes, respectively.

### Live/dead cell assays

For all experiments, the samples were examined for live and dead cells after overnight incubation using a Live/Dead Viability Cytotoxicity Kit for mammalian cells (Invitrogen MP 03224, Molecular Probes/Invitrogen). All samples used in the following experiments contained more than 99.9% live cells. All samples with less than 99.9% live cells were excluded from further analyses and recollected.

### Statistical analysis and background correction

The procedure used for background correction has been described previously by Arneth (6). The flow cytometry results were analyzed using 4-quadrant graphs in which double-positive cells were located in the upper-right (UR) quadrant, double-negative cells were present in the lower-left (LL) quadrant and single-positive cells were located in the lower-right (LR) and upper-left (UL) quadrants. The proportions of activated T-cells were determined for the 2 T-lymphocyte sub-populations, CD4+ and CD8+, using the generated graphs. The proportion of activated T-cells corresponded to the number of double-positive T-cells (UR) divided by the total number of single-positive and double-positive T-cells (UR + LR) for each of the 2 populations. Thus, the quotients q4 and q8 for the CD4+ and CD8+ T-cells, respectively, were calculated according to the following equations:

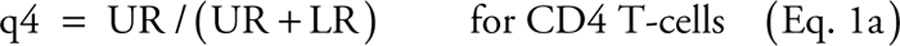


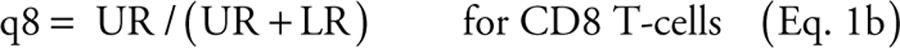

Notably, q4 was calculated from the CD69+ vs CD4+ plot, and q8 was calculated from the CD69+ vs CD8+ plot.

Untreated blood samples (Tube 2) consistently exhibited weak T-lymphocyte activation. However, the level of background activation, indicating the extent of T-cell activation in these untreated samples, differed among subjects. To account for the background activation of T-cells in each experiment, differences in the calculated quotients between the paired treated and untreated blood samples from each subject were calculated, as described by Arneth (6), using the following equations for CD4+ and CD8+ T-lymphocytes, respectively:

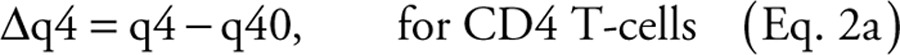


and

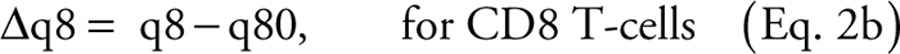

Thus, Δq4 and Δq8 represent the proportions of activated T-lymphocytes in the treated sample (q4 and q8, respectively) after subtracting the background activation of the paired untreated sample (q40 or q80, respectively).

To investigate the effects of overnight insulin or GAD incubation, the proportions of activated CD4+ and CD8+ T-lymphocytes with or without insulin or GAD treatment were determined and compared. Next, the data were statistically compared between the patients’ samples and healthy subjects’ samples using Student’s *t*-test and the Wilcoxon test (healthy subjects vs type 1 DM patients, and healthy subjects vs type 2 DM patients). For each statistical analysis, data from identical experiments (incubation with insulin or GAD) performed using both groups of samples (healthy subjects’ and patients’ samples) were assessed.

As described earlier, quantitative differences in T-lymphocyte activation were calculated for each sample between aliquots incubated with and without insulin (Δq4 and Δq8) or with and without GAD (Δq4 and Δq8). These differences constituted robust, stable experimental results.

### Sub-group analysis

The following sub-groups were analyzed:

1. Patients with type 1 DM and positive insulin autoantibodies (IAA) at the onset of the disease (*N* = 4).
2. Patients with type 1 DM and positive GAD autoantibodies at the time of the study (*N* = 4).

All patients from both sub-groups exhibited elevated CD4+ and CD8+ T-lymphocyte reactivities against both human insulin and GAD.

### Ethics and consent to participate

This study was approved by the ethics committee of Giessen University. All subjects and patients gave their consent to participate in this study after receiving the appropriate information.

## Results

**Figure 1:**
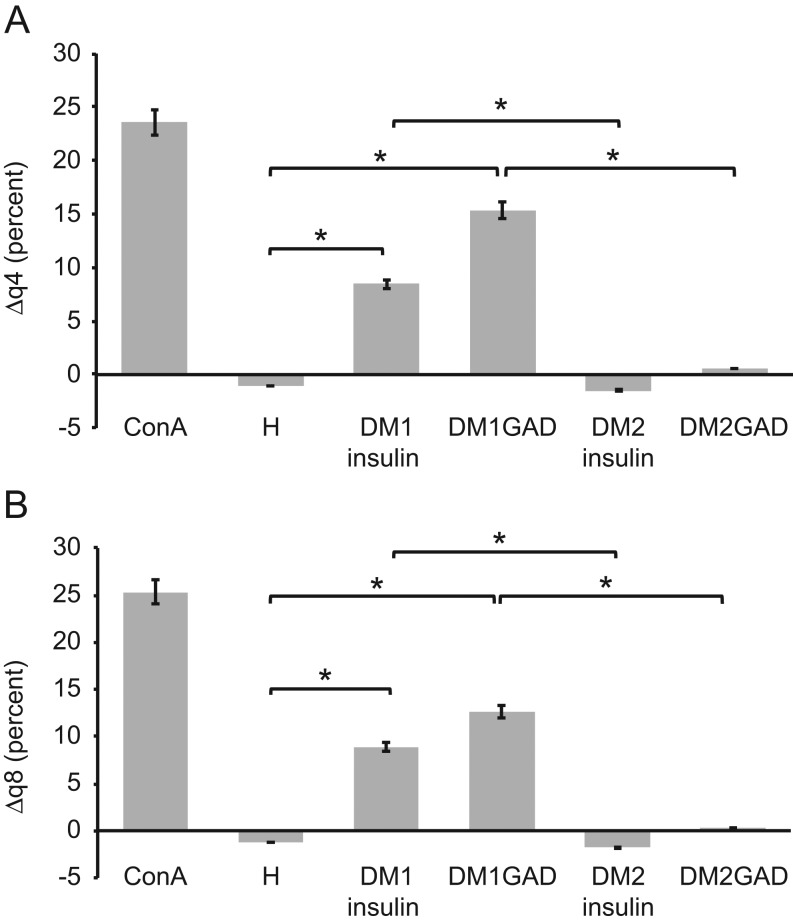
Comparisons of the populations of activated CD4+ T-cells (Δq4, A) and activated CD8+ T-cells (Δq8, B) between insulin- and GAD-treated samples and paired, untreated samples. After overnight incubation with insulin or GAD, a significant increase in the number of activated T-lymphocytes was observed in samples from type 1 DM patients compared with those from type 2 DM patients or healthy subjects (*significant difference at *P* < 0.01). Δq4: difference in CD69+/CD4+ T-cells in the tubes after pre-incubation with one of the following substances and in the respective untreated sample from the same subject. Δq8: difference in CD69+/CD8+ T-cells in the tubes after pre-incubation with one of the following substances and in the respective untreated sample from the same subject. ConA, pre-incubated with concanavalin A (positive control; DM1 GAD, patients with DM1, pre-incubation with GAD; DM1 insulin, patients with DM1, pre-incubation with insulin; DM2 GAD, patients with DM2, pre-incubation with GAD; DM2 insulin, patients with DM2, pre-incubation with insulin); H, healthy subjects.

**Figure 2:**
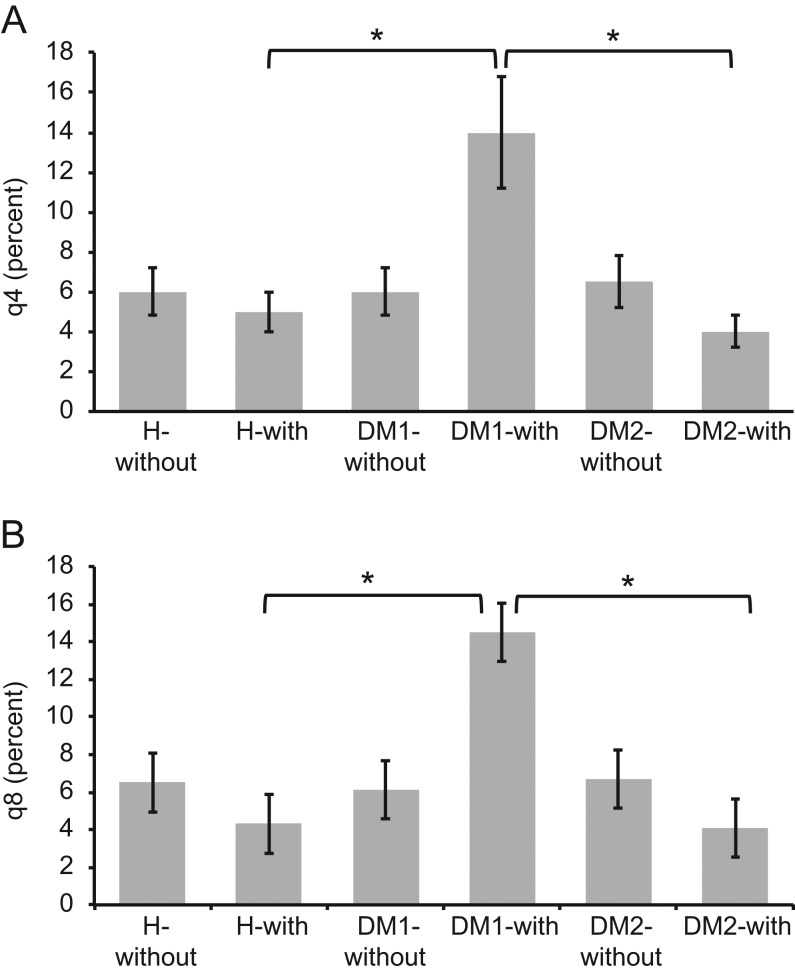
The percentages of activated CD4+ T-lymphocytes (A) and CD8+ T-lymphocytes (B) in insulin-treated and untreated samples. After overnight incubation with insulin, a significant increase in the number of activated T-lymphocytes was observed in whole blood samples from patients with type 1 DM compared with those from patients with type 2 DM or healthy subjects. In comparison, after insulin incubation, significantly fewer activated T-lymphocytes were detected in the samples from the type 2 DM patients and healthy subjects (*significant difference at *P* < 0.01). DM1-with, DM1 patients after pre-incubation with insulin; DM1-without, DM1 patients without pre-incubation with insulin; DM2-with, DM2 patients after pre-incubation with insulin; DM2-without, DM2 patients without pre-incubation with insulin; H-with, healthy subjects after pre-incubation with insulin; H-without, healthy subjects without pre-incubation with insulin; q4, percent CD69-positive CD4 T-cells; q8, percent CD69-positive CD8 T-cells.

**Figure 3:**
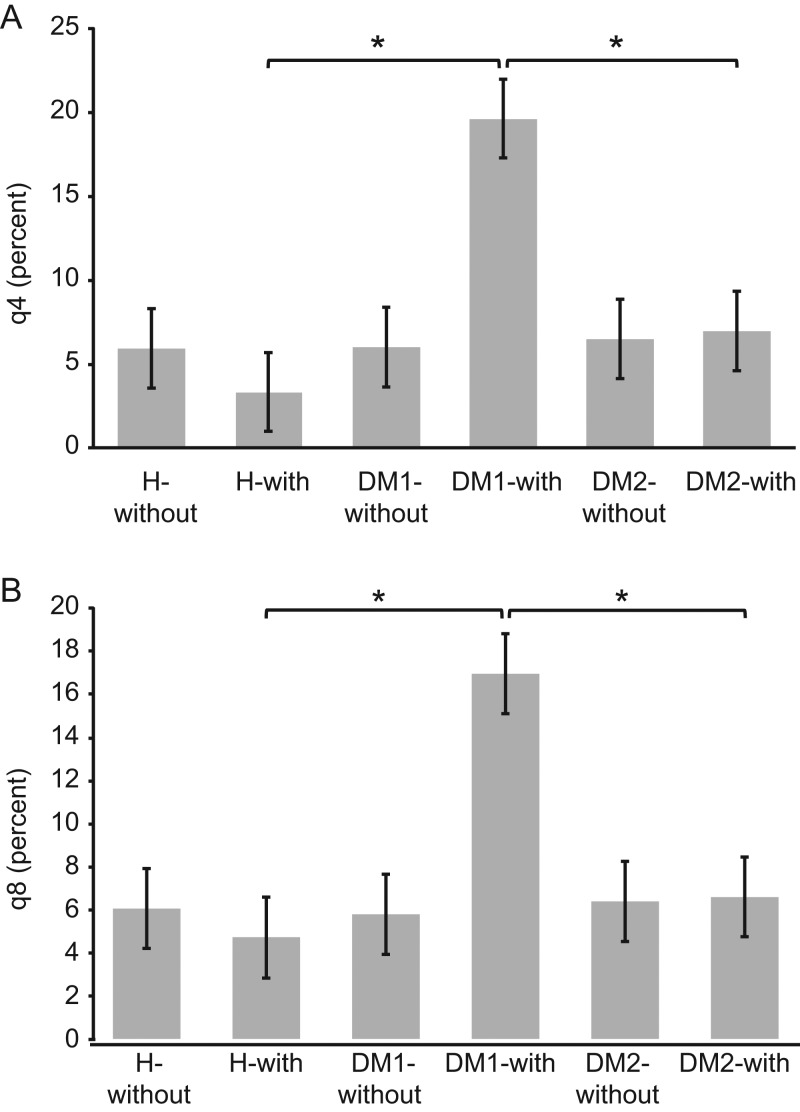
The percentages of activated CD4+ T-lymphocytes (A) and CD8+ T-lymphocytes (B) in GAD-treated and untreated samples. After overnight incubation with GAD, a significant increase in the number of activated T-lymphocytes was observed in whole blood samples from patients with type 1 DM compared with those from patients with type 2 DM or healthy subjects. In comparison, after GAD incubation, significantly fewer activated T-lymphocytes were detected in the samples from the type 2 DM patients and healthy subjects (*significant difference at *P* < 0.01). DM1-with, DM1 patients after pre-incubation with insulin; DM1-without, DM1 patients without pre-incubation with insulin; DM2-with, DM2 patients after pre-incubation with insulin; DM2-without, DM2 patients without pre-incubation with insulin; H-with, healthy subjects after pre-incubation with insulin; H-without, healthy subjects without pre-incubation with insulin; q4, percent CD69-positive CD4 T-cells; q8, percent CD69-positive CD8 T-cells.

**Figure 4:**
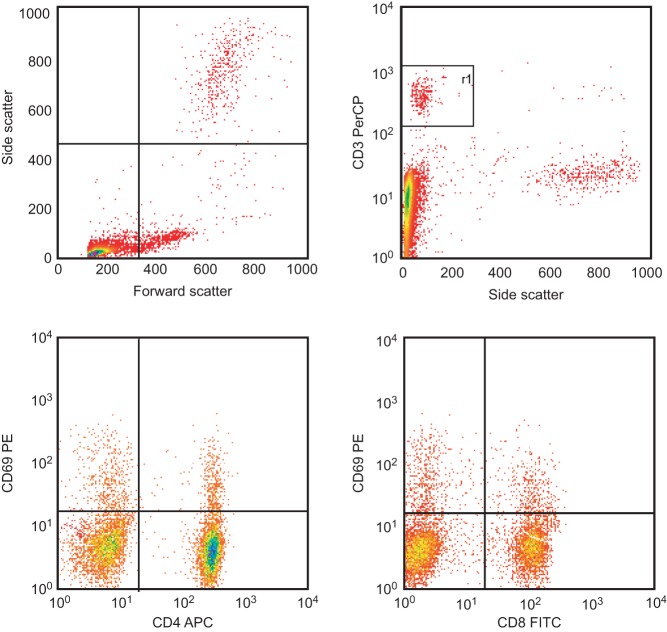
Raw flow cytometric data of a concanavalin A-pretreated whole blood sample from a healthy subject. Concanavalin A was used as a positive control.

Figure 1 presents the differences between Δq4 (Fig. 1A) and Δq8 (Fig. 1B) values for the activated cell populations from all patients and healthy subjects. Following overnight incubation with insulin or GAD, significantly higher numbers of activated T-lymphocytes were detected in the type 1 DM samples than in the type 2 DM and healthy samples. Figure 2 depicts the percentages of activated CD4+ (Fig. 2A) and CD8+ (Fig. 2B) T-lymphocytes in the insulin-treated and untreated samples. In addition, Figure 3 shows the percentages of activated CD4+ (Fig. 3A) and CD8+ (Fig. 3B) T-lymphocytes in the GAD-treated and untreated samples. Figure 4 shows the cytometric data of a concanavalin A-pretreated whole blood sample from a healthy subject.

After overnight incubation with both insulin and GAD, significant increases in the number of activated T-lymphocytes were observed in the whole blood samples from the type 1 DM patients compared with those from the type 2 DM patients and healthy subjects.

## Discussion

The peripheral blood samples from type 1 DM patients that were not treated with insulin or GAD had slightly higher numbers of activated T-lymphocytes than those from the type 2 DM patients and healthy subjects.

In contrast, after incubation with insulin or GAD, the percentages of CD69+ T-lymphocytes were significantly increased in the samples from most of the type 1 DM patients compared with those from the type 2 DM patients and healthy subjects. Thus, exposure of the samples from the healthy subjects and type 2 DM patients to insulin induced the downregulation of CD69 expression, leading to fewer activated T-lymphocytes. The CD69 expression was similar between the T-lymphocytes from the healthy subjects and the type 2 DM patients.

However, exposing samples from type 2 DM patients and healthy subjects to insulin induced the downregulation of CD69 expression, which was not affected by exposing samples from healthy subjects to GAD. In contrast, incubation of the type 1 DM patients’ samples with insulin resulted in the elevated CD69 expression, and similar results were observed following incubation of these samples with GAD. Indeed, overnight incubation of the type 1 DM patients’ samples with human insulin led to significantly increased CD69 expression on both CD4+ and CD8+ T-lymphocytes, in accordance with the results observed following overnight incubation with GAD.

It is likely that the T-lymphocyte response following a second contact with the same antigen or epitope will be stronger and more pronounced than that following the first contact (7, 8), and the results observed for the patients with type 1 DM (but not for those with type 2 DM) were indicative of prior T-lymphocyte exposure to GAD.

These results suggested that latent pre-activation of CD4+ and CD8+ T-lymphocytes occurred in a subset of the type 1 DM patients in response to epitopes on the insulin polypeptide chain. In addition, latent pre-activation of CD4+ and CD8+ T-lymphocytes toward GAD occurred in a second subset of the type 1 DM patients.

Interestingly, downregulation of CD69 expression following insulin exposure was observed in samples from both the healthy volunteers and the type 2 DM patients. However, incubation of the samples from these individuals with GAD did not cause significant changes in the CD69+ expression. As insulin is a normal physiological extracellular, serum and/or plasma protein present in both healthy subjects and type 2 DM patients, and its suppression, but not its elevation, are physiologically normal.

Insulin is a physiological serum protein, similar to albumin. Therefore, it should have a negative regulatory effect on the immune response. Indeed, the Fc termini of immunoglobulins contain effective regulatory signal sequences with immunomodulatory functions, referred to as Tregitope sequences (9). Immunoglobulins to albumin and insulin seem to contain active immunomodulatory sequences, which could prevent normal physiological serum proteins from eliciting an immune response.

The present study is the first to present a pathophysiological concept to describe the latent pre-activation of T-lymphocytes in type 1 DM patients. This novel finding is supported by the presence of IAA in a subset of the type 1 DM patients. Indeed, the presence of these autoantibodies and B-lymphocyte activity during the early stages of type 1 DM have been well documented (10, 11, 12, 13, 14).

The presence of both insulin and GAD autoantibodies in a subset of the type 1 DM patients indicated a B-lymphocyte response to insulin and/or GAD as autoantigens. In addition, the presence of pre-activated T-lymphocytes indicated that a T-lymphocyte response to insulin as a major autoantigen occurred in a portion of the type 1 DM patients and that a T-lymphocyte response to GAD occurred in a second, distinct portion of these patients.

It is unknown whether IAA in type 1 DM patients are essential for disease pathogenesis or whether their appearance represents a major step in disease development, and this topic remains controversial in the extant literature (15, 16). In addition, the presence of autoantibodies could indicate a non-specific, bystander autoimmune response (15, 16).

In type 1 DM, the autoimmune reaction is directed against insulin-producing beta cells in the pancreas. Because this reaction is required for disease onset, it is possible that immunization against insulin as a major autoantigen is responsible for the presence of self-reactive T-lymphocytes.

In the context of autoimmune disease, the outcomes of this immunization include not only formation of insulin and GAD autoantibodies in B-cells but also, as demonstrated in the present study, the latent pre-activation of T-lymphocytes by insulin as an autoantigen in T-cells, as well as by GAD.

Both insulin and GAD autoantibodies are often present before the onset of DM, and they can be used for the early diagnosis of type 1 DM in some patients. However, the present study detected ongoing pathogenic T-cell activity. Furthermore, the level of T-cell activity was even higher than the corresponding level of B-cell activity, given that the T-cell activity was detected in all patients with autoantibodies but that an additional increase was observed in the type 1 DM patients, even in those without detectable autoantibodies.

### Limitations of the study

We used a surrogate marker (CD69) in a small number of patients and controls. Therefore, the results of this study need to be repeated in a larger patient cohort to determine the benefit for patients with type 1 DM. The observed CD69 activation could be an epiphenomenon.

## Conclusions

The main findings of the present study are the latent reactivity and pre-activation of CD4+ and CD8+ T-lymphocytes toward two autoantigens associated with DM, namely insulin and GAD. In particular, T-lymphocytes specific to insulin and GAD were detected in whole blood samples from type 1 DM patients, although this pre-activation was observed in only a subset of the patients.

It is still unclear whether the latent pre-activation of T-lymphocytes toward insulin or GAD autoantigens described herein is relevant to the pathogenesis of type 1 DM. It is also possible that these observations indicate non-specific, bystander autoimmune response. However, the T-lymphocyte reactivity described herein might be useful as a marker of disease activity.

The occurrence of autoantibodies in type 1 DM has been well documented (10, 11, 12, 13, 14, 15, 16). In this disease, autoantibodies develop against a variety of antigens, including insulin (IAA) and GAD (GAD autoantibodies), all of which are localized to beta cells in the pancreas.

The findings of this study demonstrate the latent pre-activation of CD4+ and CD8+ T-lymphocytes capable of responding to insulin and/or GAD as autoantigens, and they suggest that these autoreactive cells can be detected in specific subsets of type 1 DM patients.

## Declaration of interest

The author has no competing interests that have influenced the writing of this manuscript; in particular, no specific financial interests, relationships and/or affiliations relevant to this article exist other than those listed on the title page of the manuscript.

## Funding

This work did not receive any specific grant from any funding agency in the public, commercial or not-for-profit sector.

## Authors’ contribution statement

B A performed the study and wrote the manuscript.
